# Factors associated with the intention to use maternity waiting homes among pregnant women in North Ethiopia

**DOI:** 10.1371/journal.pone.0304510

**Published:** 2024-06-13

**Authors:** Kahsay Zenebe Gebreslasie, Abrahaley Welay Beyene, Rebecca Susan Dewey, Tewelde Gebrehawerya

**Affiliations:** 1 Department of Midwifery, College of Health Sciences, Mekelle University, Mekelle, Ethiopia; 2 Departments of Midwifery, College of Medicine and Health Sciences, Axum University, Axum, Ethiopia; 3 Sir Peter Mansfield Imaging Centre, University of Nottingham, Nottingham, United Kingdom; Tribhuvan University, NEPAL

## Abstract

**Introduction:**

Maternity waiting homes are residential facilities, located near a qualified healthcare facility, where pregnant women can await their delivery and be transferred to a nearby healthcare facility shortly before delivery, or earlier if complications arise. Although evidence has shown that maternity waiting homes reduce maternal and neonatal mortality, there is limited information about factors associated with the intention to use maternity waiting homes in the study area. Therefore, the aim of this study was to identify factors associated with intention to use maternity waiting homes among pregnant women.

**Methods:**

The study used a community-based cross-sectional study design. Simple random sampling was used to select 399 pregnant women. Data were collected using an interviewer-administered pre-tested structured questionnaire.A binary and multivariate logistic regression analysis was performed.

**Result:**

Two hundred and eighty (70.2%) women indicated they intended to use a maternity waiting home during their current pregnancy. Participants’ educational status, having experienced a previous institutional delivery, the affordability of food while staying at the maternity waiting home, placing a burden on their attendant, having children in the household who can be cared for by the community or family during the woman’s absence, and having household chores covered by their family/community were the factors associated with the intention to use a maternity waiting home.

**Conclusion:**

Relatively few respondents intended to use maternity waiting homes. Empowering women and giving them agency by ensuring their needs are met are important measures necessary to increase the use of maternity waiting homes.

## Background

A maternity waiting home (MWH) is a residential facility, located near a healthcare facility, where pregnant women can await their delivery and be transferred to a nearby medical facility shortly before delivery, or earlier in the case that complications are expected. Pregnant women near their due date are recommended to stay close to a healthcare facility providing essential maternity care and/or care for obstetric and neonatal complications, to increase access to specialized healthcare for populations living in remote areas or with limited access to services [1,2].

Drivers of success in reducing maternal mortality range from making improvements at the provider and healthcare system level to implementing interventions aimed at reducing social and structural barriers. Globally, several key strategies have been implemented at the national level to reduce delays in access to these vital services. Many low-income countries have introduced MWHs. These innovative strategies help to increase institutional deliveries and consequently decrease maternal mortalities associated with delays in reaching obstetric care [3,4].

Millions of women globally still do not receive the maternal healthcare services they need to survive pregnancy and thrive. An estimated 289,000 women worldwide die each year during or shortly after pregnancy or childbirth. Most of these women live in low-income nations and the disparities among and within countries are immense. Almost all (99%) preventable maternal deaths occur in low-and-middle-income countries [5].

In high-income countries, almost all pregnant women attend at least four antenatal care visits and receive treatment from skilled healthcare workers during and after childbirth, whereas in low-income countries, only 40% of pregnant women receive the recommended antenatal care [6]. According to the Ethiopia Demographic and Health Survey (EDHS) 2016 report, Ethiopia has high pregnancy-related maternal mortality with an estimated 412 maternal deaths per 100,000 live births. The report stated that only 62% of women received antenatal care from a skilled provider at least once for their most recent birth. Despite institutional deliveries increasing from 10% in 2011 to 26% in 2016, home delivery is still high (73%) primarily in hard-to-reach areas, and as few as 17% of pregnant women and 13% of newborns received a postnatal check within the first 2 days of birth [7].

Four strategies have been proposed in Ethiopia to address the sociocultural, distance-related, and financial barriers to receiving maternity care, as these are reportedly the key barriers prohibiting mothers from accessing the available healthcare facilities. The four proposed strategies are: community mobilization, cultural adaptation of birthing services to overcome socio-cultural barriers, maternity waiting homes to overcome distance-related barriers, and conditional payments to address financial barriers [8].

A study conducted in Ethiopia reported the rate of maternal death for women who did not access a MWH to be 10 per 1000 live births, while the rate for those using MWHs was 1 per 1000 live births. Further, there is a great disparity between the rates of stillbirth among those who do and do not use MWHs. Among those not using MWHs, the risk of stillbirth was 181 per 1000 live births, while for those using MWHs this risk decreased to 17 per 1000 live births [9].

Studies conducted in Zambia and Liberia reported that, compared to respondents from healthcare centers without MWH access, those from centers with MWH access expressed similar levels of intention to use an MWH for a future pregnancy (98.8% and 98%, respectively). A study reporting facility-based cross-sectional data collected in Jimma reported that 38.7% of mothers surveyed had previously used an MWH, and that 57.3% intended to use an MWH in the future. A community-based cross sectional study conducted in Southern Nations, Nationalities and Peoples, southwestern Ethiopia, indicated that only 7.0% women were aware of MWHs prior to the survey. After learning about the existence of MWHs, 55.1% of women indicated they would be likely to use one during their current or next pregnancy [10–13].

Previous studies have explored the factors associated with the intention to use an MWH. A study conducted in southern Ethiopia revealed that the most important predictors of the intention to use an MWH were having experienced complications during previous deliveries and anticipating relatively few barriers to using an MWH. Barriers that negatively affect a woman’s willingness to use an MWH were the need to be away from home, leaving existing children at home in the care of others, transport costs, and the burden on their attendant. According a facility-based cross-sectional study conducted in Jimma assessing the intention to use MWHs, having past experience of using an MWH, of giving birth in a healthcare institution and having more than four ANC visits were predictors of the intention to use an MWH [10,13–15].

A study characterizing barriers to using MWHs showed that transportation, cultural problems, distance, lack of awareness, and poor provider interaction prevented mothers from using MWHs [16]. A similar study also revealed that living over 30 minutes away, their wealth being in the fourth quintile, having a good perception of MWHs, and perceiving the existence of few barriers were the key predictors of using an MWH [17].

This study aims to provide baseline information on the factors associated with the intention to use an MWH with the ultimate aim of improving maternal and child survival and health. These findings will contribute to understanding and interpreting the outcomes of ongoing research examining the determinants of MWH use among disadvantaged women. The objective of this study was to identify factors associated with the intention to use MWHs among pregnant women in Wereda Tahtay Koraro (T/Koraro), North West Tigray, Ethiopia.

## Methods

### Study setting and design

Wereda T/Koraro is one of six Wereda in the North West zone of the Tigray Region of Ethiopia. T/Koraro is bordered on the southwest by Asigede Tsmbla, on the north by Laelay Adyabo, and on the southeast by Medebay Zana. The town of IndaSelassie is the administrative capital of the Wereda.

Based on the 2007 national census conducted by the Central Statistical Agency of Ethiopia, the total population of T/Koraro was projected to be 78,311 in 2018, of which 38,795 were male and 39,516 female [18]. T/Koraro has 14 administrative Kebeles and 4 healthcare centers. The total number of pregnant women in T/Koraro numbered 2,586 during first half-year report produced in the 2018–19 administrative period [19]. A community-based cross sectional study design was used and the study was conducted between 1^st^ and 30^th^ April, 2019.

### Study population and sampling procedures

The study population comprised all pregnant women living in Wereda Tahtay Koraro. Pregnant women residents of the selected Kebeles were eligible to participate in the study. A single population proportion formula [n = (z α/2)2 p (1-p) /d2] was used to calculate the sample size, making the following assumptions: an effect size taken from a previous study conducted in southern Ethiopia reporting that 55.1% of women intended to use an MWH [13]; a 95% confidence level; a 5% margin of error; and a 5% of non-response rate. Consequently, a sample size of 399 pregnant women was required. Wereda T/Koraro comprises 14 Kebeles. Using a lottery method, five Kebeles were selected from the 14 Kebeles. The sample size of 399 was proportionally allocated to each Kebele based on the number of pregnant women resident in each kebele, as obtained from health extension records of each Kebele prior to data collection, using the rule of proportionate sampling of the ratio of the number of pregnant women in the Kebele relative to the population size (p = 399/1095 = 0.364, where, 399 is the calculated sample size, 1095 is total number of pregnant women in the selected Kebeles, to give the proportion, p) to ensure proportionality. This resulted in multiplying the number of pregnant women in each selected Kebele by 0.364 to achieve the number of pregnant women in that kebele who would be invited to participate in the study. Finally, simple random sampling was used to select the registration numbers of the required number of pregnant women fulfilling the inclusion criteria in each Kebele.

### Data collection tool and procedures

The questionnaire was adapted from published literature and translated into Tigrigna (the local language) and then back into English to ensure internal consistency. A pilot study was conducted in a sample of 20 (5%) pregnant women in a different Wereda of the same zone, Wereda Asgede Tsmbla, Mayhanse Kebele using the same methodology. Following this pilot, minor amendments were made to the survey wording and skip patterns to address issues that arose during the pilot study. Five midwives and nurses collected the data by conducting face-to-face interviews using a structured questionnaire. The supervisor and study principal investigator monitored the data collection process to ensure the completeness and consistency of survey response data. Training was given to the data collectors and supervisor to ensure they were aware of the study objective and relevance, the need to ensure confidentiality of participant data, participants’ rights, the informed consent process, and interview techniques.

### Data analysis procedures

After data collection, completed questionnaires were coded. Data were entered into EpiData version 4.2 and analyzed using SPSS version 23. Data cleaning was performed to check for frequencies, accuracy, and consistencies and missed values and variables. Descriptive statistics, tables, and graphs of frequencies, means, standard deviations, and percentages were used to present the data. A bivariate logistic regression model analysis was conducted to assess the association between the explanatory and outcome variables. The multivariate logistic regression analysis used only variables exhibiting associations of p ≤ 0.2 in the bivariate analysis. Adjusted odds ratios (AORs) with 95% confidence intervals (CIs) were used to measure the strength of associations between dependent and independent variables. Statistical significance was considered at a threshold of p < 0.05.

### Ethical considerations

Ethical approval was obtained from the institutional review board of Mekelle University college of health sciences. An official letter of cooperation was sent to the Tigray regional health bureau from the college’s ethical committee. A letter of cooperation was sent to each participating Kebele’s administrative health office. Following an explanation of the purpose of the study, written consent was obtained from participants. Participants were informed that they were free to withdraw consent and discontinue participation at any time without consequences for their healthcare. Participants were assured of the confidentiality of the information provided and of the privacy of their participation. They were also informed that all data obtained from them was kept confidential through the use of participant codes instead of using any personal identifiers and that such data would only be used for the purpose of the study and putted in secured place.

## Results

### Socio-demographic characteristics

A total of 399 pregnant women participated with a response rate of 100%. The mean (± standard deviation; SD) age of the respondents was 32.6±5.0 years. The majority (97.5%) of respondents were Orthodox Christian, with the remaining 10 (2.5%) being Muslim. Almost all (97.7%) were married, while 7 (1.8%) were divorced and 2 (0.5%) were widowed. The vast majority (99%) were Tigrian in ethnicity and the rest (1%) were Amara (Table 1).

**Table 1:** Socio-demographic characteristics among pregnant women of Wereda Tahtay Koraro, northwest Tigray, Ethiopia, May 2019.

Variable	Category	Frequency	Percentage
**Age**	<25	33	8.3
	25–29	72	18.0
	>29	294	73.7
**Educational status**	Unable to read & write	154	38.6
	Able to read & write	129	32.3
	Primary and higher	116	29.1
**Occupation**	Farming	339	85.0
	Government employed	15	3.8
	Merchant	31	7.8
	Student	14	3.5
**Husband’s occupation**	Government employed	20	4.5
	Farmer	330	82.7
	Merchant	41	10.3
	Student	4	1.0
	Daily laborer	4	1.0
**Husband’s educational status**	Unable to read & write	102	25.6
	Able to read & write	190	47.6
	Primary and higher	107	26.8
**Monthly income (ETB)**	<448	35	8.8
	448–641	60	15.0
	641–843	46	11.5
	843–1259	118	29.6
	>1259	140	35.1
**Religion**	Orthodox Christian	389	97.5
	Muslim	10	2.5
**Marital status**	Married	390	97.7
	Divorce	7	1.8
	Widowed	2	0.5
**Ethnicity**	Tigrian	395	99
	Amhara	4	1

### Obstetric characteristics

The majority of the pregnant women (82.7%) had attended at least one antenatal care visit and only 19 (4.8%) had attended four or more visits. Concerning participants’ obstetric characteristics, 341 (85.5%) of the respondents were multigravida, while 58 (14.5%) were primigravida. While most (85.5%) respondents had given birth before, 263 (65.9%) had given birth to their last child in a healthcare institution, while 78 (19.6%) had given birth at home (Table 2).

**Table 2:** Obstetric characteristics of pregnant women of Wereda Tahtay Koraro, North West Tigray, Ethiopia, June 2019.

Variable	Category	Frequency	Percent
**Attended an antenatal care visit**	Yes	330	82.7
	No	69	17.3
**Number of antenatal care visits**	1^st^ visit	76	23
	2^nd^ visit	140	42.42
	3^rd^ visit	95	28.8
	4^th^ visit	19	5.6
**Parity**	Primigravida	58	14.5
	Multigravida	341	85.5
**Place of birth before current pregnancy**	Home	78	19.6
	Healthcare institution	263	65.9

### Awareness and utilization of MWHs and physical accessibility

Only 233 respondents (58%) were aware of MWHs prior to the study, with 39 (9.8%) having had previous experience of using an MWH, and 200 (50.1%) being aware that their nearest healthcare facility was equipped with an MWH. Most (87.7%) respondents had traveled longer than 60 minutes to their nearest healthcare facility to access the service (Table 3).

**Table 3:** Awareness and utilization of maternity waiting home and physical accessibility of pregnant women of Wereda Tahtay Koraro, North West Tigray, Ethiopia, June 2019.

Variable	Category	Frequency	Percent
**Aware of MWHs**	yes	233	58.4
	No	166	41.6
**Previous experience of using an MWH**	Yes	39	9.8
	No	360	90.2
**Aware about nearest MWH**	Yes	200	50.1
	No	199	49.9
**Distance to nearest health facility**	>30 minute	350	87.7
	< = 30 minute	49	12.3

### Intention to use a maternity waiting home

**Fig 1:**
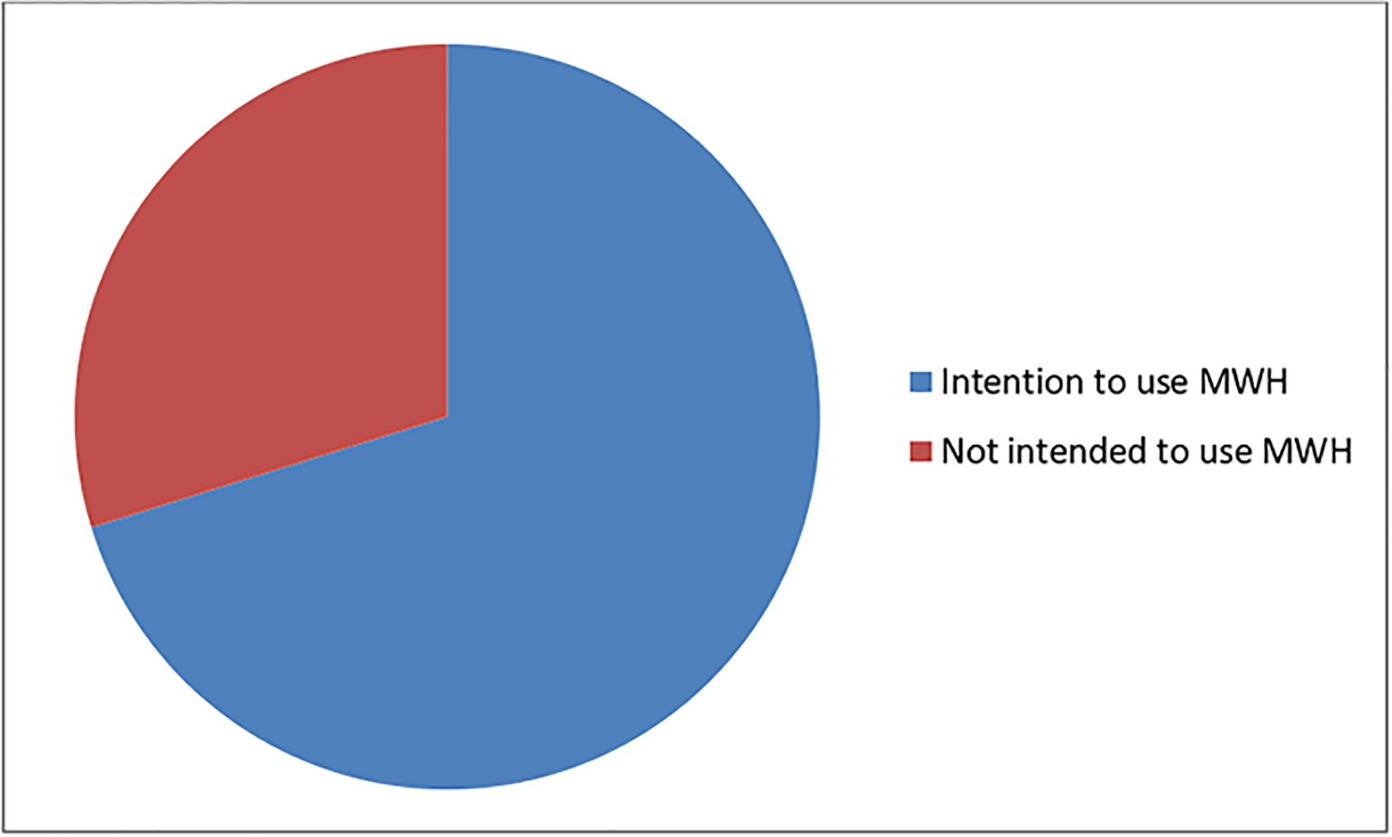
Intention to use a maternity waiting home (MHW) of pregnant women of Wereda Tahtay Koraro, North West Tigray, Ethiopia, June 2019.

After being informed about the existence of MWHs, 280 (70.2%) respondents indicated they intended to use an MWH during their current pregnancy, while 119 (29.8%) did not intend to do so (Fig 1).

### Factors associated with intention to use an MWH

The results of the bivariate regression analysis showed that the following covariates had associations (p < 0.2) with the intention to use an MWH: participant age, participant educational status, husband’s educational status, having attended at least one antenatal care visit, the place of their previous delivery, affordability of food while staying at the MWH, the ability to bring an attendant, having children at home cared by others, having a person to take care of household chores, being away from work (separate from household chores), and the burden of requiring their attendant to be away from other work/obligations. Consequently, these factors were entered into the multivariate logistic regression analysis to control for confounding factors.

In the multivariate logistic regression, while controlling for confounding factors, the following factors were significantly (p < 0.05) associated with the intention to use an MWH: participant’s educational status, the place of their previous delivery, affordability of food while staying at MWH, the ability to bring an attendant, having children at home cared by others, and having a person who take care of household chores. Respondents who were able to read and write were 2 times more likely to intend to use an MWH (AOR = 2.113 95%CI; 1.022, 4.366) compared to those unable to read and write. Respondents who had completed primary and higher education were 4 times more likely to intend to use an MWH (AOR = 4.017 95% CI; 1.516, 10.645) compared to those who were unable to read and write. Women who had given birth to a previous child in a healthcare institution were 2.6 times more likely intend to use an MWH (AOR = 2.582 95%CI; 1.217, 5.478) than those who gave birth at home.

The likelihood of intending to use an MWH was higher among pregnant mothers who could afford food while staying at the MWH (AOR 4.560 95% CI; 2.380, 8.736). Those who considered it possible for a family or community member to go with them to the MWH as an “attendant” were 2.8 times more likely to express the intention to use an MWH (AOR = 2.779 95% CI; 1.322, 5.840) compared to those who considered it impossible. Those who were not dependent upon family or community members to take care of household chores during their absence were more likely to intend to use an MWH (AOR = 4.374 95% CI; 2.154, 8.881) compared to those who needed to rely on someone for this. Respondents who were able to find someone to take care of their children at home during their absence were 3.8 times more likely to express the intention to use an MWH (AOR = 3.78695% CI; 1.626, 8.8140) compared to their counterparts (Table 4).

**Table 4:** Multivariate analysis factors associated with intention to use a maternity waiting home among pregnant women in North West Tigray, Ethiopia, June, 2019.

Intention to use a maternity waiting home						
Variable		Yes	No	COR(95%CI)	AOR(95%CI)	P-value
**Participant’s educational status**	Unable to read & write	89 (57.8)	65(42.2)	1	1	
	Able to read & write	93 (72.1)	36 (27.9)	1.887(1.144,3.112)	2.113 (1.022, 4.366)	.043
	Primary and higher	98 (84.5)	18 (15.5)	3.976(2.192,7.214)	4.017(1.516, 10.645) 11.089)	.005
**Previous place of delivery**	Home	45(57.7)	33(42.3)	1	1	
	Healthcare institution	186(70.7)	77(29.3)	1.771(1.051,2.985)	2.582 (1.217, 5.478)	.013
**Food while staying at MWH**	Not affordable	62(47.7)	68(52.3)	1	1	
	Affordable	218(81.0)	51(19.0)	4.688(2.960,7.425)	4.560 (2.380, 8.736)	.000
**Ability to bring an attendant**	Not possible	107(53.0)	95(47.0)	1	1	
	Possible	173(87.8)	24(12.2)	6.400(3.849,10.64)	2.779 (1.322, 5.840)	.007
**Having children at home cared for by others**	Not possible	90(48.9)	94(51.1)	1	1	
	Possible	141(89.8)	16(10.2)	9.204(5.090,16.63)	3.786(1.626,8.8140)	.002
**Having someone to take care of household chores**	Not possible	77(45.8)	91(54.2)	1	1	
	Possible	203(87.9)	28(12.1)	8.568(5.206,14.10)	4.374 (2.154, 8.881)	.000
**Attendant being away from other work / obligations**	Not possible	161(63.9)	91 (36.1)	1	1	
	Possible	119(81.0)	28(19.0)	2.402(1.479,3.903)	.811(.353, 1.860)	.620

## Discussion

There is limited literature reporting factors associated with the intention to use MWHs in the Tigray region. Consequently, this study aimed to identify factors associated with the intention to use MWHs among pregnant women living in Wereda Tahtay Koraro. In this study, 70.2% of women reported the intention to use an MWH during their current pregnancy. Participants’ educational status, place of their previous delivery, the affordability of food while staying at the MWH, the feasibility of bringing an attendant during their stay, having children at home cared by others, and having someone to take care of household chores in their absence were factors found to be significantly associated with the intention to use an MWH.

This finding that 70.2% of pregnant women intended to use an MWH is lower than those of studies conducted in Zambia and Liberia reporting that 98% and 98.8%, respectively, of study participants expressed the desire to use an MWH for future pregnancies [11,12]. These differences may be due to differences in the study designs and settings. For instance, the study conducted in Liberia was facility-based, whereas the present study was community based. However, the present finding was higher than that of a study conducted in Jimma district which reported an intention rate of 57.3%, and southern Ethiopia which reported an intention rate of 55.1% [10,13]. These differences may be due to the study period, as the present study was conducted in in 2019 in contrast to the studies conducted in Southern Ethiopia (2014) and Jimma (2016). The elapsed time between studies may account for increased awareness of recent improvements in maternal and child health services and enhanced healthcare-seeking behavior among pregnant women.

Pregnant women who were able to read and write were 2 times more likely to express an intention to use MWH services compared to those who were unable to read and write. This ability to read may help pregnant women to understand information distributed by health extension workers and other healthcare professionals in the form of leaflets and manuals, which in turn may lead to increasing their knowledge in relation to maternal and child health. Pregnant women who had completed primary and higher education were 4 times more likely express an intention to use an MWH compared to those who are unable to read and write. These findings contradict those of a study conducted in Attat hospital, southern Ethiopia [20]. This may be due to the study design and period, as the study conducted in Attat retrospectively accessed hospital records from 2011 to 2014. These differences could also be due to the first MWH in Ethiopia being commissioned at Attat hospital, and both governmental and non-governmental guidelines being strictly followed for its implementation, making the initiative well known to all mothers including those from remote areas with little access to education. However, the present findings are in line with those of a study conducted in southern Ethiopia, eastern Gurage Zone [13], potentially due to educated women in the region being easily able to understand information provided in the form of health education by health professionals and health extension workers. Pregnant women who gave birth to their last child in a healthcare institution were more likely to express the intention to use an MWH than those who gave birth at home. This is consistent with the findings of a study conducted in Jimma zone that revealed giving birth in a healthcare institution to be a predictor of the intention to use an MWH [10]. This could be due to health education given postnatally following previous births outlining the potential threats of delivering at home, with the result of raising awareness of the benefits of delivering in a healthcare facility and staying at an MWH.

This study also revealed that pregnant women who considered it possible for a family or community member to go with them to the MWH to act as an “attendant” were 3 times more likely to express the intention to use an MWH than those who considered it impossible. This is also in agreement with the findings of a study conducted in Eastern Gurage, southern Ethiopia [13]. This could be due to the traditional occupations of women in the rural areas of Ethiopia placing a greater burden on women, such as farming or taking care of cattle, such that they might find it difficult to find another person to accompany them when staying at an MWH.

Another finding of this study is that pregnant women who were not dependent on family or community members to take care of household chores during their absence were more likely to intend to use an MWH compared to their counterparts. A possible explanation for this the greater burden on women living in rural areas to take care of household chores while the husband’s responsibility is that of farming. Consequently, women often prioritize household chores over their own wellbeing or the ability to stay in an MWH, given that they may need to be absent for four weeks or more before delivery, unless someone else can share in this burden. This study also revealed that women who were able to organize childcare for their existing children at home during their absence were more likely to express the intention to use an MWH compared to their counterparts. This finding is in agreement with those of studies conducted in Zambia, a rural health center in Ethiopia, Jimma district, and Eastern Gurage [10,11,13,21]. This may be due to the commonly held societal belief that women, especially those living in rural areas, carry the burden of the childcare responsibilities. Consequently, unless the pregnant woman is able to find someone to take care of her children at home during her absence, childcare responsibilities prohibit her from staying at an MWH.

Finally, this study found that the likelihood of expressing an intention to use an MWH was higher among pregnant women who could afford food while staying at an MWH. This is consistent with the findings of studies conducted in a rural health center of Ethiopia and Jimma district [10,21]. This could be due to an inability find a family member or attendant who can provide them with a continuous supply of food, often requiring travelling significant distances, during their stay at an MWH. This may also be due to the pregnant woman’s economic situation prohibiting access to food during their stay.

## Conclusion and recommendation

The study revealed that few respondents intended to use maternity waiting homes. The educational status of participants, previous institutional delivery experience, affordability of food while staying at the MWH, placing a burden on an attendant, having other children in the household cared for by the community or family during the pregnant woman’s absence, and having household chores covered by family members or the community were factors found to be significantly associated with the intention to use an MWH. Policy makers such as the ministry of health are recommended to consider the significant impact of MWH use in reducing maternal mortality. They should focus on efforts to empower women through education and offering encouragement to women to consider delivering at a health facility. Regional health bureaus should provide food for women staying in MWHs.

### Study limitations

Data were collected using interviewer-administered questionnaires. Interviewees’ responses may have been influenced by the interviewer’s presence and social desirability bias in self-reporting the intention of whether or not to use an MWH. Due to the decision not to use the design effect when conducting the sample size calculation, the resulting sample size may be underpowered.

## Supporting information

S1 File (XLS)
